# Surgical Management among Patients with Spontaneous Supratentorial Intracerebral Haemorrhage Admitted in a Tertiary Care Centre: A Descriptive Cross-sectional Study

**DOI:** 10.31729/jnma.7178

**Published:** 2022-08-31

**Authors:** Dinuj Shrestha, Upama Sharma, Janam Shrestha, Gopi Nepal, Bishal Shrestha, Pranaya Shrestha, Samir Acharya, Pritam Gurung, Resha Shrestha, Sudan Dhakal, Pravesh Rajbhandari, Basant Pant

**Affiliations:** 1 Department of Neurosurgery, Annapurna Neurological Institute and Allied Sciences, Maitighar, Kathmandu, Nepal

**Keywords:** *intracerebral haemorrhage*, *mortality*, *surgical procedure*

## Abstract

**Introduction:**

Spontaneous intracerebral haemorrhage is the second most common form of stroke and the most deadly one. An understanding of changing trends in the epidemiology of intracerebral haemorrhage prevalence, its risk factors, current practice in management, case fatality, and longterm outcome is essential to measure the effectiveness of stroke prevention and various treatment efforts. The objective of this study was to find out the prevalence of surgical management among patients with spontaneous supratentorial intracerebral haemorrhage in a tertiary centre.

**Methods:**

A descriptive cross-sectional study was conducted in the Department of Neurosurgery from January 2017 to December 2019. Ethical approval was obtained from the Institutional Review Committee (Reference number: 06/2020/IRC-ANIAS). A convenience sampling method was used. Data of the patients were retrieved from online medical records. Point estimate and 95% Confidence Interval were calculated.

**Results:**

Among 221 patients with spontaneous supratentorial intracerebral haemorrhage, 115 (52.04%) (45.45-58.63, 95% Confidence Interval) underwent surgical management. In-hospital mortality was seen in 23 (20%) and survivors at 3 months were 78 (67.82%) patients.

**Conclusions:**

The prevalence of surgical management among spontaneous supratentorial intracerebral haemorrhages was higher than in other studies done in a similar setting.

## INTRODUCTION

Intracerebral haemorrhage (ICH) is the most disabling and least treatable form of stroke even with the advancement in medical knowledge.^1,2^ To date no therapy has shown proven benefit in improving outcomes after spontaneous ICH, decreasing either its death rate or the burden of a long-term disability.^3^ Hypertension correction, osmotherapy, and early hemostatic therapy have no strong effects.^3,4^ Regarding the role of surgery for spontaneous ICH, the largest randomised, multicentered international surgical trial for intracerebral haemorrhage (STICH) could not show conclusive results and failed to establish any standard international guidelines.^5^

From our part of the world, we have a very limited number of studies and data regarding the surgical management of ICH. Adequate data on prevalence are required to identify the burden of the disease, baseline characteristics, and its risk factors and to establish guidelines and preventable measures.

This study aimed to find the prevalence of surgical management among patients with spontaneous supratentorial intracerebral haemorrhage admitted in a tertiary care centre.

## METHODS

This was a descriptive cross-sectional study conducted in the Department of Neurosurgery, Annapurna Neurological Institute and Allied Sciences, Kathmandu, Nepal. Data of the patients were retrieved from online medical records from January 2017 to December 2019. Ethical approval was obtained from the Institutional Review Committee (IRC) of the same Institute (Reference number: 06/2020/IRC-ANIAS). Cases that had surgery for spontaneous ICH with a Glasgow coma scale of 4 or above were included in the study. Patients who had ICH secondary to vascular malformation, traumatic ICH, purely intraventricular haemorrhage, or infratentorial haemorrhage were not included in the study. A convenience sampling method was used. The sample size was calculated by using the following formula,


$$\begin{array}{ll} \text{n} & ={\text{Z}}^{2}\times \frac{\text{p}\times \text{q}}{{\text{e}}^{\text{2}}}\\ & =1.96^{2}\times \frac{0.50\times 0.50}{0.07^{2}}\\ & =196 \end{array}$$

Where,

n= minimum required sample size
Z= 1.96 at 95% Confidence Interval (CI)
p= prevalence of taken as 50% for maximum sample size calculation
q= 1-p
e= margin of error, 7%

The calculated sample size was 196. However, we have included 221 patients. The patients who underwent surgical management were identified to retrieve data from medical records. Data regarding the demographic profile, associated risk factors, Computed tomography (CT) scan findings, and surgical method were recorded. Results were categorised into functional outcome (good outcome and poor outcome) as per the Glasgow outcome scale (GOS) at discharge and 3 months duration. GOS >3 as good outcome where GOS ≥ 3 as poor outcome.^6^

Data were entered in Microsoft Excel 2013 and analysed in IBM SPSS Statistics 20.0. Point estimate and 95% Confidence Interval were calculated.

## RESULTS

Among 221 cases of spontaneous supratentorial ICH, surgical management was done in 115 (52.04%) (45.45-58.63, 95% CI). The mean age of the patient was 54.60±13.45 years. Of all the ICH cases, 64 (55.7%) patients were known hypertensive (Table 1).

**Table 1:** Demographic profile of the patients (n= 115).

Variables	n (%)
**Gender**	
Male	80 (69.56)
Female	35 (30.43)
**Alcohol consumption**	37 (32.17)
**Smoker**	32 (27.82)
**On anticoagulants**	2 (1.73)
**Comorbidities**	
Hypertension	64 (55.65)
Diabetes mellitus	9 (7.82)
Coagulopathy	2 (1.73)
Amyloid angiopathy	5 (4.34)

Among the known hypertensive patients, a majority 37 (57.81%) were non-compliant with antihypertensive medications (Table 2).

**Table 2:** Hypertension status (n= 115).

Variables	n (%)
Known hypertensive	64 (55.70)
Unknown hypertensive	48 (41.73)
Normotensive	3 (2.60)

Vomiting was present in 55 (47.81%) of patients, 17 (14.82%) had a seizure and anisocoric pupil was seen in 11 (9.40%) of patients at the time of presentation. A total of 37 (57.81%) were noncompliant to medication. CT characteristics and associated findings are illustrated (Table 3).

**Table 3:** CT findings among patients with spontaneous supratentorial ICH (n= 115).

Variables	n (%)
**Laterality**	
Left	57 (49.56)
Right	58 (50.43)
**Site of haemorrhage**	
Putamen	74 (64.34)
Parietal	21 (18.26)
Thalamic	9 (7.82)
Frontal	5 (4.34)
Temporal	4 (3.47)
Occipital	2 (1.73)
**Characteristics findings**	
Perilesional edema	90 (78.26)
Intraventricular extension	54 (46.95)
Midline shift	54 (46.95)
Subarachnoid haemorrhage	12 (10.43)
Hydrocephalus	11 (9.56)

External ventricular drain (EVD) was kept in 8 (6.95%) patients, 12 (10.43%) underwent minimally invasive stereotactic evacuation of the hematoma. None of the patients required decompressive craniectomy (Table 4).

**Table 4:** Various interventions (n= 115).

Variables	n (%)
Craniotomy	103 (89.56)
Stereotactic	12 (10.43)
External ventricular drain	8 (6.95)
Decompressive craniectomy	-

In-hospital mortality was 23 patients (20.00%) and overall survivors were 78 (67.82%) at 3 months. The remaining 14 (12.17%) patients died between discharge and 3 months follow-ups duration.

Among the survivors, 69 (88.46%) patients were living with good outcomes and 9 (11.53%) were poor outcomes in terms of GOS at 3 months duration. Overall, among all the 115 patients, 69 (60%) had a good outcome and 46 (40%) had a poor outcome at 3 months. A total of 55 (54.5%) patients who were categorised as poor outcomes at the time of discharge had improved and progressed to good outcomes at 3 months duration (Table 5).

**Table 5:** Outcome at discharge and 3 months follow-up period (n= 115).

Outcome at discharge		Outcome at 3 months	
**Good**	**Poor**	**Good**	**Poor**
**(GOS>3)**	**(GOS≤3)**	**(GOS>3)**	**(GOS≤3)**
14 (12.17)	101 (87.82)	69 (60.00)	46 (40.00)

## DISCUSSION

The prevalence of surgically managed cases among spontaneous supratentorial ICH was 52.04%. In the literature review, 7-16% of patients get operated on for spontaneous ICH, which is less as compared to our study.^7^ One of the studies from Finland done between 1985-1991 reported surgical management among ICH cases in only 6% of patients.^8^ Total of 20% of ICH patients who entered the registry between 1999 and 2001 underwent surgical treatment as part of the Japan Standard Stroke Registry Study (JSSR).^9^

The rationalisation of performing surgery is to prevent secondary brain injury from mass effect, cerebral hernia and raised intracranial pressure (ICP),^10^ as well as improve the environment of peri-hemorrhagic tissues by augmenting cerebral perfusion and reducing neurotoxic substances.^11^

Two larger randomised controlled trials, STICH^12^ and STICH II,^13^ neither of them showed significant differences between early surgery versus best medical treatment, but even these trials had limitations because of patients selection criteria and a large number of patients being cross-over between treatment groups. In addition, two meta-analyses have reported a slight but significant benefit of surgical treatment over the best medical management.^14,15^

In our study, the overall survivor was 78 out of 115 patients (67.82%), among the survivors 69 (88.46%) patients are living with good functional outcomes in terms of GOS at 3 months duration. There were 55 (54.45%) patients who were categorised as having poor outcome at the time of discharge but progressively improved to favourable functional outcome in 3 months. Senior authors from Nepal also reported a 60% progression of outcome from unfavourable to favourable in 3 months follow up when compared at the time of discharge in patients operated for spontaneous ICH.^16^ Similar outcome was also reported from a hospital of Pakistan with 22% in-hospital mortality and 77.8% survivor.^17^ Our result was comparable to similar study done in Sweden concluding combination of surgery, neurocritical care and rehabilitation can result in favourable functional outcome.^18^

In subgroup analysis, it is seen that mortality is very less (5.2%) in patients with superficial bleed when compared to deep bleed (27%). Another important observation was that there were no differences in outcome and overall mortality when operated between the right and left side of ICH. This is in contrast to the conclusion of earlier studies which showed differences in functional outcomes depending upon the laterality of stroke lesion.^19,20^

Various literature has shown that untreated hypertension is an important risk factor for hemorrhagic stroke and approximately one-fourth of it would be prevented if all hypertensive patients were being treated.^21,22^ Awareness regarding hypertension, the need for its proper management, and its consequences were higher in high-income countries when compared with low-income countries like Nepal.^23^ Even within a country, the unknown and neglected hypertension were higher in the rural populations compared with the urban.^24^ Our study also shows that majority of the cases were either unknown hypertensive (41.73%) or hypertensive patients who are not taking medications regularly (57.81%). Through this study, we would also like to highlight that it is a very concerning, major public health issue that is unseen, neglected, and contributing to uncontrolled hypertension. There is still a taboo regarding taking antihypertensive medication in many parts of our country. In addition, there is no clearly defined national plan and policy for hypertension prevention and management in Nepal.^25^

This study was based on a single centre and the data was collected from the medical record section using convenience sampling. So, this might not be generalizable to other hospitals.

## CONCLUSIONS

The prevalence of surgical management among spontaneous supratentorial ICH is higher than in other studies done in similar settings. The lack of proper guidelines and protocol regarding the management could have an impact on this higher prevalence. Neglected hypertension could be a major public health problem contributing to a large portion of intracerebral haemorrhage and could be prevented.
